# Coronary artery bypass grafting associated to aortic valve replacement in the elderly: survival and quality of life

**DOI:** 10.1186/1749-8090-7-13

**Published:** 2012-02-06

**Authors:** Mariano Vicchio, Marisa De Feo, Salvatore Giordano, Raffaella Provenzano, Maurizio Cotrufo, Gianantonio Nappi

**Affiliations:** 1 Department of Cardio Thoracic Surgery Second University of Naples Monaldi Hospital Naples 80100 - Italy; 2 Department of Cardiovascular Surgery Pineta Grande Hospital Castel Volturno Caserta 81030 - Italy

**Keywords:** Heart valve replacement, mechanical prostheses, elderly, quality of life, CABG

## Abstract

Myocardial ischemia is often associated to aortic valve stenosis in the elderly. Aim of this study was to evaluate the impact on survival and quality of life of CABG associated to aortic valve replacement in the septuagenarians and octogenarians.

Between January 1991 and January 2010, 520 patients ageing > 70 years underwent aortic valve replacement with a mechanical prosthesis in two Institutions. They were divided into 2 groups: Group A included 406 patients undergoing isolated aortic valve replacement; Group B 114 patients receiving aortic valve replacement and CABG. A comparative analysis of long-term survival and quality of life (SF-36 test) was performed.

Mean age was 74.2 ± 3.6 years (74.3 ± 3.6 in Group A, 74 ± 3.3 in Group B; *p *= 0.33). Hospital mortality was 9.5% (46 patients). Twenty-nine (7.8%) in Group A and 17 in Group B (15.2%)(p = 0.019). Actuarial survival was 88.5% ± 0.015 at 1 year, 81.9% ± 0.02 at 5 years, 76.6% ± 0.032 at 10 and 57.3 ± 0.1 at 15 years. Ten-year survival was 77% ± 0.034 in Group A and 77.8% ± 0.045 in Group B (p = 0.2). Multivariate analysis did not reveal associated CABG as a predictor of long term mortality. The scores obtained in the SF-36 test were similar in the two groups and significantly higher than those of the general population matched for country, age and sex (p < 0.001 in all domains).

Associated CABG determines a significant increase of hospital mortality in the elderly undergoing aortic valve replacement. Survivors did not show differences in long-term outcome and quality of life according to the presence of associated CABG.

## Introduction

Aortic valve replacement (AVR) is an accepted therapy for septuagenarians or octogenarians affected by aortic valve disease [1]. It has been demonstrated that isolated AVR can be performed with a low operative mortality rate in elderly patients, improves long-term survival and quality of life (QOL) [2]. Calcified aortic stenosis is frequently associated to coronary arteries diseases and CABG is required during the AVR procedure. Aim of the present study was to investigate the impact of CABG associated to AVR on short and long term outcome and quality of life in elderly patients.

## Material and methods

### Patient Population

Our Institutions Ethics Committee approved this study and waived the need to obtain patient consent. Between January 1991 and January 2010, 520 patients older than 70 years underwent consecutive AVR with mechanical bileaflet prostheses; by policy, mechanical devices were preferred at our Institutions in those years even in elderly patients unless life-long anticoagulation was contraindicated. In our previous studies, the incidence of anticoagulation-related complications was very low in patients older than 70 years, who underwent mechanical prosthetic implant and whose dicumarol therapy was strictly followed at our dedicated outpatient clinic [3-5].

In the light of our experience with mechanical valve implantation and with the management of oral anticoagulation in septuagenarians and octogenarians, associated to high long-term survival rates in those series showed, we prefer the use of bileaflet prostheses in elderly, with the aim to avoid the risk of a possible reoperation in the eight and ninth decade of life for structural degeneration of bioprosthesis.

The study population was divided in group A (406 isolated AVR) and Group B (114 CABG associated to AVR).

### In-hospital Management

All patients underwent preoperative full cardiological screening including trans-thoracic or trans-oesophageal echocardiography and coronary angiography. All operations were performed by the same group of surgeons. Cardiopulmonary bypass methods were uniform throughout the study: systemic moderate hypothermia was employed in all cases, along with myocardial protection through crystalloid hyperkaliemic cardioplegia infusion into the aortic root in case of normal aortic valve competence, or directly into the coronary ostia in case of aortic valve regurgitation, associated to topical cooling with cold saline.

Postoperative anticoagulant therapy was performed with oral sodium warfarin in all patients, and INR was routinely checked daily during the postoperative hospital stay period, then weekly through the first postoperative month, and subsequently on indication of our dedicated anticoagulation outpatient clinic (by default once every three or four weeks). For patients with bileaflet mechanical prostheses in aortic position the target INR ranged between 2.0 and 3.0 before 2000, and between 1.8 and 2.5 thereafter.

### Follow-up

The method of follow-up for our retrospective studies, in patients wearing mechanical valve prostheses has been described in previous reports (2-5). Follow-up has mainly been conducted on all hospital survivors during our institutional ambulatory activities and was 98.3% complete. Adverse events, with particular focus on bleeding, transient ischemic attacks, reversible or non-reversible ischemic neurologic deficits, are classified following the Guidelines for Reporting Morbidity and Mortality After Cardiac Valvular Operations as proposed by the Society of Thoracic Surgeons [6]. When appropriate, an echocardiogram follows physical examination.

To the purpose of the present analysis, data regarding the operation and the early postoperative period were retrospectively collected by hospital charts and outpatient charts review, while follow-up data of hospital survivors derived mainly from our ambulatorial activities. The anticoagulation results, the data regarding every complication possibly occurred and the scores obtained at the SF-36 test for each patient were retrospectively inserted in an electronic database. INR variability (percentage of values outside the therapeutic range) was calculated for each patient. All patients had been seen at our outpatient clinic and/or followed-up by telephone interview at least once since their discharge. In particular, of the 438 hospital survivors, 328 (74.8%) used to perform periodic controls at our ambulatory (median of the maximum number of visits per year: 13, range 12-25) and by June 2010 had already undergone a recent visit (within the last 2 months). Of the other 110 patients (48 of whom used to attend satellite regional anticoagulation centers that follow the same anticoagulation protocol as ours), 84.6% were contacted by telephone and then visited in our outpatient clinic to update their follow-up. Thus, all in all, 318 hospital survivors were visited between April and June 2010 (96.9% completeness of clinical follow-up).

#### Quality of Life Assessment

Follow-up included the assessment of perceived QOL through the SF-36 [7], which has an established validity and reliability [8]. The SF-36 questionnaire consists of 36 items grouped into eight domains:

• Physical Functioning (10 items) indicates level of limitations in lifting, bending, kneeling, or walking moderate distance.

• Role-physical (4 items) measures the degree in performing of usual activities for age and social status, such as job and community activities.

• Bodily Pain (2 items) represents the intensity, frequency, and duration of bodily pain and limitations in normal activities due to pain.

• General Health (6 items) is a measurement of perceived overall health, including past and present health.

• Vitality (4 items) measures feeling of energy, fatigue, and tiredness.

• Social Functioning (2 items) indicates ability to develop and maintain mature social relationships.

• Role-emotional (3 items) measures personal feeling of job performance at work or other activities.

• Mental Health (5 items) measures the emotional, cognitive, and intellectual status of the patient.

All SF-36 domains are scaled from 0 to 100 points, with higher scores indicating better-perceived QOL. The mean scores obtained by the two study groups were compared with those of the general Italian population matched for age and sex.

### Statistical Analysis

Continuous data were expressed as mean ± SD, and compared using Student's *t *test. Discrete variables were compared using the chi-square test. Factors significantly associated with adverse outcomes were introduced in a multivariate logistic regression model to identify independent predictors of hospital and long-term mortality. Kaplan-Meier actuarial analyses of survival rates and of freedom from valve-related complications were performed. Scores obtained in each of the 8 domains of the SF-36 test were compared with those reported [6] for the age and sex-matched Italian population. A difference yielding a *p *value of < 0.05 was considered statistically significant. SPSS statistical software, version 13.0 was employed for analysis.

Kaplan-Meier actuarial analyses of survival rates and incidence of valve-related complications were performed..

Actuarial rates are expressed as percentage of patients who were event free.

Continuous data are reported as mean ± SD. All statistical analyses were performed with SPSS version 13.0

## Results

Preoperative characteristics of the study population are reported in Table 1. Study population was composed by 520 patients who underwent AVR for aortic valve stenosis. Of them 114 (21.9%) patients received associated CABG to AVR (group B), while 406 (78.07%) received isolated mechanical valve implantation in aortic position (group A).

**Table 1:** Preoperative characteristics of the study population.

	Group A (406 pts)	Group B (114 pts)	*p*
Age	74.3 ± 3.6	74 ± 3.3	0.33

Sex (male)	173 (42.6%)	73 (64%)	0.0001

Class NYHA			

IV	36 (9%)	11 (9.6%)	0.9

III	264 (65%)	74 (64.9%)	0.93

II	106 (26.1%)	29 (25.4%)	0.97

Hypertension	258 (63.5%)	71 (62.3%)	0.9

Chronic Obstructive Pulmonary Disease	87 (21.4%)	25 (21.9%)	0.98

Diabetes	79 (19.4%)	53 (46.8%)	0.0001

Chronic Renal Insufficiency	13 (3.2%)	8 (7%)	0.12

Atrial fibrillation	31 (7.6%)	10 (8.7%)	0.85

EuroScore logistic	7.52% ± 2.18	10.4% ± 3.4	0.0001

All patients were older than 70 years with a mean age of 74.2 ± 3.6, 74.3 ± 3.6 in group A versus 74 ± 3.3 in group B (p = 0.33).

At time of surgery 47 patients (9,1%) were in New York Heart Association functional class IV, 338 (65%) were in class III, the remaining 135 (25,9%) were in class II. No difference was evident in proportion of NYHA class comparing the two groups of study population.

In-hospital death occurred in 46 patients (9.5%), 29 (7.8%) patients in group A and 17 (15.2%) in group B (p = 0.019) with statistical difference at univariate analisys (p = 0.027). Multivariate analysis on the contrary did not reveal associated CABG as a predictor of hospital mortality. The causes of death were perioperative acute myocardial infarction in 9 cases (19.6%), low output syndrome in 5 patients (10.9%), stroke in 2 (4.3%), pulmonary infection in 16 (34.8%), multi-organ failure in 10 (21.8%), malignant arrhythmia in 2 (4.3%), visceral ischemia in 2 (4.3%).

Mean follow-up was 4.2 ± 3.3 years (median 3.2 years) and ranged from 6 months to 17.7 years (2016.95 pts/years). During the follow-up time, 36 late deaths occurred (7.6% of hospital survivors). Causes of death in the follow-up were sudden death in 4 patients, myocardial infarction in 1, intracranial hemorrhage in 1, ischemic stroke in 1, neoplasm in 9, respiratory failure in 8, cirrhosis in 3, dementia in 2, postoperative death (after re-operation for prosthetic endocarditis) in 1, oldness and progressive general delay in 6. Overall actuarial survival (including hospital mortality) was 88.5% ± 0.015 at 1 year, 81.9% ± 0.02 at 5 years, 76.6% ± 0.032 at 10 years. Ten-year survival was 77% ± 0.034 in Group A, 77.8% ± 0.045 in Group B (log rank = 0.2). A multivariate analysis for long-term mortality did not reveal that associated CABG was a predictor. Dialysis-dependent renal failure was found as predictor of long term mortality (OR 22; IC 4.4-110).

### Anticoagulation-related and valve-related complications

During the follow-up period, the mean INR in this series was 2.15 ± 0.16. Four major hemorrhagic events occurred in 4 patients in the follow-up, including 2 cases of gastric bleeding (requiring surgery in 1 cases), 1 intracranial bleeding and 1 hematuria requiring hospitalization. Freedom from bleeding was 99.7% ± 0.003 at 1 year, 98.4% ± 0.008 at 5 and 96.9% ± 0.013 at 10 years. Thromboembolic complications were observed in 3 patients: 1 ischemic stroke, 1 transient ischemic attack, 1 pulmonary embolism. Freedom from thromboembolism was 99.7% ± 0.003 at 1 year, 99% ± 0.006 at 5 years and 10 years.

Freedom from valve-related complications was 99.1% ± 0.005 at 1 years, 95.5% ± 0.013 at 5 and 94.7% ± 0.015 at 10 years. Ten-years freedom from valve-related complication in the two subgroups did not show significant difference (88.5% ± 0.063 in Group A, 91.9% ± 0.034 in Group B *p *= 0.26).

### Quality of life results

**Figure 1:**
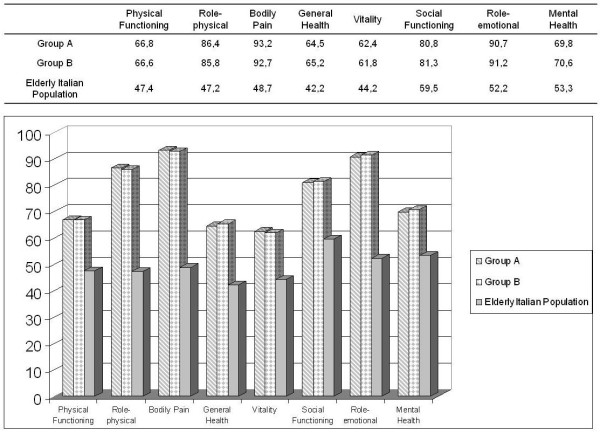
**SF-36 scores obtained in the 8 domains of the questionnaire in the Group A, in the Group B and in the Italian age and sex-matched population**.

All survivors answered the SF-36 questionnaire, and there was no statistical difference in the scores obtained in the two groups of the study population evaluating the eight domains of the test. When compared with the mean scores of the general Italian population matched for age and sex, significantly higher scores were reached by the two groups of the study population, with a *p *value of < 0.001 in all domains. The scores obtained in the various domains for Group A, Group B and the general Italian population of Elderly were reported in Figure 1.

## Discussion

The increased life expectancy of the Western population in the last decades [9], associated with the improvements in surgical standards and postoperative care, has progressively expanded the age criteria of operability in cardiac surgery [10]. As a consequence, the number of elderly patients undergoing heart valve replacement has been increasing, as well as their long-term survival. In particular it has been rising the number of elderly requiring AVR for severe calcific aortic valve stenosis, which is a tipical disease of the older age.

The growing number of elderly referring to cardiac surgical procedures has increased the attention on the outcomes in this subset of patients and on the prediction of long term survival.

When coronary artery obstructive disease is associated to severe aortic valve calcific stenosis in the elderly patients, CABG has to be combined to AVR.

Many clinical series investigating determinants of early mortality following AVR, identified associated CABG as independent predictor [11,12].

The 4^th ^European Association for Cardiothoracic Surgery Adult Cardiac Surgery 2010 database reports that overall mortality for isolated valve surgery is 3.7% while for CABG combined with valve procedures it increases to 6.2% [13].

Also in our series the association of CABG to AVR demonstrated to increase hospital mortality (15.2% vs 7.8%) and univariate analysis revealed CABG as a predictor of early death.

At the same time CABG associated to AVR improves long term survival with acceptable morbidity and mortality in elderly patients [12].

This data is confirmed by several reports: on the arena of long term survival, Akins et al. Reported the survival of patient following CABG is essentially equivalent to that of age and gender matched cohorts from the general population up to 5 years after surgery [14]. Sergeant and associated demonstrated that the actuarial survival of elderly coronary artery by-pass patients came actually be better than that predicted for the general population out to 10 years, whereas survival of the young coronary by-pass patients is poorer than a comparable cohort from the general population [15].

Findings of our study confirmed that long term mortality and morbidity are not significantly different when CABG is associated to AVR in elderly patients suffering of severe calcified aortic valve stenosis and obstructive coronary disease.

In order to extend the investigation to long term quality of life, the SF-36 test was applied in both groups of patients. Group A and Group B showed similar scores in QOL that resulted better of age and gender matched population. The increased life expectancy results in a growing necessity to maintain maximum functioning and independent lifestyle. Health-related QOL is a multidimensional concept based on the patient's perception of his or her health and integrates not only physical functioning but also psychologic status and social dimensions. Standardized questionnaires, especially those self-completed by patients, are a practical, efficacious, and inexpensive method of collecting data. There is a growing interest in the use of health status to evaluate clinical strategies, and because improvement in QOL is considered to be one of the principal goals of valve surgery [16], methods of QOL assessment are increasingly adopted in the clinical research in this field. From reports on postoperative QOL, patients deciding among treatment options may value information about the change in QOL that they can expect after valve surgery. Therefore, QOL needs to be assessed in large and well-defined patient subsets, and it is particularly important to evaluate QOL in elderly patients, who have a higher prevalence of comorbidity, a more severe surgical stress, and a higher risk of postoperative complications, all factors that may hinder improvement in QOL. Although numerous methods exist for evaluating QOL of patients [17], the validated SF-36 questionnaire [8] is comprehensive yet concise, can be completed in 10 to 15 minutes, and can be administered in person, by phone, or by mail, even in elderly patients [7].

Investigators focusing on long-term outcomes after AVR in octogenarians have reported a positive impact on QOL [18]. Sundt and colleagues described postoperative SF-36 scores in AVR patients aged older than 80 years that were comparable with those of the general elderly population [19]. In the present study, we obtained in seven of the eight domains of the test significantly higher scores than the mean general Italian population matched for age and sex.

When interpreting this result, it should be considered that more than 70% of the patients in our study population were in NYHA functional class III to IV before the operation, so a high percentage of our patients experienced, for a various period of time, a moderate to severe limitation to their daily activity. Symptom relief and the return to previous lifestyle can probably increase the perception of a patient's own health status. Similarly, the Italian general population scores were lower than in our study population because the healthy elderly are prone to compare their current physical and psychologic performances with their youth, with a perceived difference caused by the effects of the aging process itself. Other authors in series of AVR [20] found similar differences between the previously operated on elderly and the age-matched control population, in particular for what concerns the social functioning and emotional domains.

Associated CABG determines an increase of hospital mortality in the elderly undergoing aortic valve replacement; at the same time survivors did not show differences in long-term outcome and quality of life according to the presence of associated CABG.

## Competing interests

The authors declare that they have no competing interests.

## Authors' contributions

MV has ideated the research and has written the manuscript, while MDF and SG have conducted the clinical follow-up. RP has interrogated all patients about the quality of life questionnaire. MC and GN have performed surgical interventions. All authors read and approved the final manuscript.
